# Self-esteem instability and affective instability in everyday life after remission from borderline personality disorder

**DOI:** 10.1186/s40479-020-00140-8

**Published:** 2020-11-24

**Authors:** Philip S. Santangelo, Tobias D. Kockler, Marie-Luise Zeitler, Rebekka Knies, Nikolaus Kleindienst, Martin Bohus, Ulrich W. Ebner-Priemer

**Affiliations:** 1 Mental mHealth Lab, Karlsruhe Institute of Technology, Karlsruhe, Germany; 2 Institute of Psychiatric and Psychosomatic Psychotherapy, Central Institute of Mental Health, Medical Faculty Mannheim, Heidelberg University, Mannheim, Germany

**Keywords:** Borderline personality disorder, Remission, Self-esteem instability, Affective instability, Unstable symptomatology, E-diary, Ambulatory assessment, Ecological momentary assessment

## Abstract

**Background:**

Borderline personality disorder (BPD) is defined by a pervasive pattern of instability. According to prior findings and clinical theories, self-esteem instability and affective instability are key features of BPD. Previous e-diary studies showed that instability in self-esteem is heightened and that it is highly intertwined with affective instability in BPD in comparison to healthy controls (HC). The present study sought to extend these findings by adding symptomatologically remitted BPD patients (BPD-REM), i.e. former patients with BPD who met four or fewer BPD criteria within the past year, as a comparison group.

**Methods:**

To examine differences regarding self-esteem instability and affective instability, we used e-diaries for repeatedly collecting data on self-esteem, valence, and tense arousal 12 times a day for four consecutive days while participants underwent their daily life activities. Determining three different state-of-the-art instability indices and applying multilevel analyses, we compared 35 BPD-REM participants with previously reported 60 acute BPD patients (BPD-ACU) and 60 HC.

**Results:**

Our results revealed that self-esteem instability was significantly lower in the BPD-REM compared to the BPD-ACU group, irrespective of the instability index. In contrast, there were no significant differences regarding affective instability between the BPD-REM participants and those in the BPD-ACU group. The comparison between the BPD-REM with the HC indicated both a significantly higher instability in self-esteem as well as significantly heightened affective instability in the BPD-REM participants. Moreover, even though the associations were not significant, we found tentative support for the assumption that affective changes that are accompanied by changes in self-esteem are experienced as more burdensome and negatively impact the quality of life of remitted BPD participants.

**Conclusions:**

This study builds on growing evidence for the importance of self-esteem instability in BPD. Whereas affective instability has been reported in various psychiatric disorders and might indeed constitute a transdiagnostic marker of affective dysregulation, our results indicate that self-esteem instability might be a specific symptom that construes the unique pathology in BPD.

## Background

Borderline personality disorder (BPD) is commonly characterized by instability in emotion and mood, self-image and identity, interpersonal relationships, as well as impulse and behavioral control [1]. Accordingly, several classification criteria for BPD define symptomatology by its problematic fluctuations and its burdensome ups and downs [1]. Any investigation of the temporal fluctuations of these symptoms comes with a specific challenge: Time must be considered in the assessment and the analyses to capture the specific dynamic element. Previous studies often relied on self- or rater-based retrospective reports of perceived instability. However, several empirical studies give rise to reasonable doubts on whether people are capable to report past dynamics of unstable symptoms correctly. Namely, these studies revealed that the congruence between the actual ups and downs of affect (obtained by repeated assessments of momentary states) and retrospective assessments of affective instability (obtained by using interviews or questionnaires) is modest at best (e.g. [2]). The rationale behind the critics is that retrospective measures presumably assess subjective (i.e. mental) representations of an experience but not the experience itself (e.g. [3]). In contrast, electronic diaries (e-diaries) offer the possibility to repeatedly assess symptoms of interest in real-time and in the real world. This results in multiple momentary ratings without retrospective biases and with high ecological validity that can be used to model unstable symptomatology and dynamic within-person processes [4]. Thus, e-diaries can indeed track experiences to investigate dynamic within-person processes. In the past, e-diaries have been successfully used to examine symptom dynamics in daily life in patients with BPD (see [5] for a review). However, most e-diary studies focused on affective instability, while e-diary studies to examine self-image and self-esteem in patients with BPD are scarce. This is despite the fact that there is copious empirical evidence that unstable self-esteem is associated with multiple BPD-like symptoms in healthy subjects’ everyday lives: Self-esteem instability has been found to be associated with diminished self-concept, self-concept clarity, and lower self-acceptance [6, 7]. Individuals with unstable self-esteem are more reactive to daily events [8–10] and have a greater tendency to experience anger, hostility, and aggressive outbursts [11, 12]. Moreover, they have a greater tendency to engage in maladaptive coping styles [13] and maladaptive interpersonal behaviors [7] and cognitions [14], and to have suicidal ideations [15]. Thus, self-esteem instability has been associated with low psychological adjustment and poor functioning in healthy subjects’ everyday lives.

Two e-diary studies in subclinical individuals found that those with high levels of BPD features showed higher levels of self-esteem instability and affective instability compared to individuals with low levels of BPD features [16, 17]. In line with these studies in subclinical BPD samples, two recent studies brought new attention to the importance of self-esteem in BPD. Santangelo et al. [18] empirically showed the highly intertwined interplay of self-esteem instability and affective instability in 60 patients with BPD and 60 healthy controls (HC) using a high sampling frequency e-diary protocol with hourly assessments over four days. In detail, the study showed heightened instability in self-esteem and affective instability across various statistical indices in BPD patients compared to the HC participants. Analyzing the pattern of self-esteem instability revealed large decreases in self-esteem, particularly in states of high self-esteem, and only small increases in states of low self-esteem, suggesting sudden dramatic worsening and slow recovery of self-esteem in patients with BPD. The importance of self-esteem instability was furthermore delineated since only self-esteem instability significantly predicted general, BPD-specific, and depressive psychopathology when self-esteem instability and affective instability were entered simultaneously as predictors in multiple regressions.

A recent study examined the role of within- and between-person effects of self-esteem and affective state in predicting dysfunctional behavior [19]. A total of 119 patients with BPD carried e-diaries to repeatedly report their current self-esteem, emotional valence, tense arousal, and whether they engaged in dysfunctional behaviors, in hourly intervals 12 times a day for four consecutive days. Dynamic structural equation modeling revealed that, on the within-person level, high momentary negative affect and, on the between-person level, low trait self-esteem, mainly predicted dysfunctional behaviors. Namely, results showed that high momentary negative affect (i.e. negative valence and high tension) predicted upcoming dysfunctional behaviors, whereas the higher a person’s trait level of self-esteem the less likely it was that this person engaged in dysfunctional behavior in general. In addition, we also found that low momentary self-esteem predicted dysfunctional behavior and that higher trait levels of negative affect predicted engaging in dysfunctional behaviors, though to a lesser extent. Taken together, these studies highlight the significance of self-esteem in BPD. This is especially important since it has been hypothesized that the lack of specificity of affective instability for BPD, when compared to clinical control groups might be explained by the highly intertwined temporal interplay of affective and self-esteem instability [18]. Namely, previous e-diary studies failed to show specificity of affective instability for BPD empirically. Peculiarly, contrary to the expectations of Santangelo et al. [20], patients with posttraumatic stress disorder and patients with bulimia nervosa showed similarly heightened affective instability as patients with BPD. Neither global instability nor likelihood of extreme changes nor in-depth analyses of the patterns of affective states did differentiate between BPD patients and the clinical control groups. Similarly, investigations trying to demonstrate specificity in BPD using subcomponents of affective dysregulation [21], emotional granularity [22], and emotion sequences [23] also failed, with only minor differences regarding frequencies and intensities of specific emotions [24]. Santangelo et al. [20] suggested that affective changes that are accompanied by changes in self-esteem might be experienced as more burdensome and threatening. Since changes in self-esteem are more prevalent in BPD in general and, therefore, are often related to frequent changes in affect, overall affective changes might be experienced as more devastating in patients with BPD.

Regarding remission in BPD, problems relating to the personality structure and organization can continue even when patients achieve symptomatic remission, i.e. drop below the diagnostic threshold on the continuum of BPD criteria [25]. Findings across prospective multi-wave follow-up studies of BPD suggest that diagnostic continuity is moderate to low and that personality psychopathology decreases over time [26–29]. However, differential levels of stability across BPD features have been reported; that is, some diagnostic criteria have been shown to be more stable than others. Affective instability was most prevalent at baseline, and even with the number of patients fulfilling the criterion declining, it was the most prevalent of the BPD criteria over ten years of follow-up [28]. In contrast, the follow-up assessments in two year intervals indicated a more pronounced decline in identity disturbance compared to affective instability, especially in the first four years, whereas both symptoms showed similar trajectories afterward. In the Collaborative Longitudinal Personality Disorders Study [28], about half of the patients who fulfilled the criterion of affective instability at the baseline assessment did not fulfill it at the four year follow-up. In contrast, about two-thirds of patients who fulfilled the criterion of unstable sense of self at the baseline assessment did not fulfill it at the four year follow-up. Similarly, whereas after ten years, only about 20% of patients still fulfilled the criterion of unstable sense of self, about 40% of patients still fulfilled the criterion of affective instability. In a similar vein, McGlashan et al. [30] showed in the McLean Study of Adult Development that after two years, only about 50% of patients still fulfilled the criterion of unstable sense of self, whereas about two-thirds of patients still fulfilled the criterion of affective instability. Furthermore, Zanarini et al. [31] showed that the median time for remission for identity disturbance is two to four years, whereas the median time for remission for affective instability is four to six years. Taken together, these studies examining the course of individual symptoms of BPD demonstrated an overall decrease in all symptoms, but with the diagnostic criterion of unstable sense of self remitting more frequently and at a quicker rate than the criterion of affective instability.

Regardless of the strong focus in the literature on clinical remission rather than personal recovery, several studies addressed recovery from BPD. Those studies using a multifaceted definition of recovery, i.e. attainment of social and vocational competence in addition to symptomatic remission, consistently revealed a pattern of improved psychopathology and persisting impaired psychosocial functioning over an extended period of time (for an overview see [32]). Even though many patients with BPD exhibit fewer symptoms as time progresses, psychosocial functioning remains impaired, and only part of the patients attain social and vocational competence after symptomatic remission (e.g. [28, 33, 34]). These studies demonstrated that psychosocial functioning often remains impaired with only low to modest improvement over both 10-year follow-up [28, 33] as well as 16-year follow-up [34] indicating severe and persistent impairment in psychosocial functioning. Being not able to function in one’s desired life role and valued activities also impacts overall satisfaction with life. Thus, patients with BPD showed only minor improvements in satisfaction with life over an extended period of time [33]. Among the strongest predictors associated with poorer global outcomes and satisfaction with life in patients with BPD were experiencing affective symptomatology [32] as well as self-esteem [35]. Therefore, the burdensomeness of the interplay of self-esteem instability and affective instability may negatively affect patients’ level of functioning and quality of life.

Taken together, previous findings indicate that recovery from BPD combining both symptomatic remission and good psychosocial functioning seems difficult to attain for many patients with BPD. Despite increases in functioning, the level of functioning is still indicative of ongoing difficulties in patients with BPD. Thus, the good psychosocial functioning needed to achieve a good global outcome is rarely attained and retained among remitted patients with BPD. Therefore, BPD has a significant impact on the quality of life, even after a loss of diagnosis.

### Aims

The aim of this study was to investigate further the instability in self-esteem and affect in everyday life in BPD. Previous e-diary studies have shown heightened self-esteem instability and affective instability in patients with BPD compared to healthy control participants [18] as well as in subclinical individuals with high levels of BPD features [16, 17]. For a better understanding of the symptomatology, it is of paramount interest whether self-esteem instability and affective instability are indicative of clinical group belonging, i.e. whether they differ between individuals with a current BPD disorder, remitted disorder, and healthy controls. The present study aimed to extend prior findings utilizing e-diaries with a high sampling frequency (i.e. hourly assessments over four days) in 35 BPD patients with a symptomatic remission (BPD-REM). We compared instability in self-esteem and affective instability in the BPD-REM participants to the sample reported in Santangelo et al. [18], i.e. 60 patients with acute BPD (BPD-ACU) and 60 healthy controls (HC). We also tested whether changes in affect that are accompanied by changes in self-esteem are experienced as more burdensome and threatening. We sought to shed more light on the impact of the association between affective changes and changes in self-esteem on participants’ ability to function in their life roles and activities, which also impacts the overall quality of life. Therefore, we examined whether the strength of the association between affective changes and changes in self-esteem is related to the level of functioning and self-rated quality of life in the BPD-REM participants.

## Methods

### Participants and procedures

We assessed 35 symptomatically remitted patients with BPD (BPD-REM) and compared them with the participants reported in Santangelo et al. [18], i.e. 60 patients with acute BPD (BPD-ACU) and 60 healthy controls (HC). Thus, a total of 155 female participants between 18 and 64 years of age (mean age of 31.15 ± 9.89 years) was analyzed in this study. All patients in the BPD-ACU group met the DSM-IV diagnostic criteria for BPD [1], whereas all participants in the BPD-REM group constituted participants with a symptomatic remission, i.e. participants who met less than five BPD criteria according to DSM-IV within the past year. Patients with acute BPD were recruited from the waiting list for a residential dialectical behavior therapy treatment program [36] at the Central Institute of Mental Health Mannheim in Germany. The HC were randomly selected from the national resident register of the City of Mannheim. Further details on the recruitment of the BPD-ACU and the HC group are reported in Santangelo et al. [18]. The 35 remitted patients with BPD constitute a subsample of the 58 participants enrolled in the study by Zeitler et al. [35]. Zeitler et al. [35] contacted former patients with BPD 12 to 18 years after taking part in one of two dialectical behavior therapy treatment studies at Freiburg University in Germany [37, 38]. All these patients had initially been diagnosed as meeting BPD criteria using standardized diagnostic instruments. Further details are reported in Zeitler et al. [35]. Only participants with a symptomatic remission or loss of diagnosis (i.e. who met four or less BPD criteria according to DSM-IV within the past year) were enrolled in the current e-diary study. Of the 35 BPD-REM participants, *n* = 9 fulfilled none of the BPD criteria, whereas *n* = 11 fulfilled one, *n* = 5 fulfilled two, *n* = 7 fulfilled three, and *n* = 3 fulfilled four diagnostic criteria for BPD at the time of enrollment in the study. The most prevalent BPD criteria in the BPD-REM participants were “stress-related paranoia or dissociation” (*n* = 13, i.e. 38%), followed by “suicidal and self-harming behavior” and “affective instability” (each fulfilled by *n* = 12, i.e. 34%), and “identity disturbance, unstable self” (*n* = 6, i.e. 17%). All the other diagnostic criteria were fulfilled by only a small number of BPD-REM participants (≤ *n* = 3, i.e. ≤ 9%). It is important to note that both the BPD-ACU patients and the BPD-REM participants were acquired before (BPD-ACU) or after (BPD-REM) treatment on specialized units for dialectical behavior therapy treatment that were run by a team around Martin Bohus, who implemented dialectical behavior therapy treatment in Germany.

### Psychiatric diagnoses

In all groups, axis I disorders were assessed using the German version of the Structured Clinical Interview for DSM–IV axis I Disorders (SCID–I [39]). In the BPD-ACU and the HC group, axis II disorders were assessed using the Structured Clinical Interview for DSM–IV axis II Disorders (SCID–II [40]). Participants in the BPD-REM group underwent a thorough diagnostic procedure to assess current DSM-IV BPD criteria using the International Personality Disorder Examination (IPDE [41]). Postgraduate psychologists administered all three well-validated diagnostic instruments with very good psychometric properties (e.g. SCID–I kappa = .71, SCID–II kappa = .84, IPDE kappa = .80 [42, 43]). The exclusion criteria for enrollment in the study differed by group. In the BPD-ACU group, patients with a history of schizophrenia, bipolar disorder, or current substance use disorder were not enrolled in the study. For the HC group, individuals with any current or past axis I or axis II diagnoses were excluded from the study. The exclusion criteria for the BPD-REM group were acute intoxication with alcohol or drugs and current psychotic, manic, or severe depressive episodes. Whereas the age of the participants in the BPD-ACU and the HC group was restricted to ranging from 18 to 46 years, no restrictions regarding age were applied in the BPD-REM group. Table 1 provides sample characteristics by group. A Kruskal-Wallis H test revealed significant differences regarding age in the three groups, with subsequent Mann-Whitney-U tests indicating that the participants in the BPD-REM group were significantly older compared to both the BPD-ACU and the HC participants (Table 1; BPD-REM vs. BPD-ACU: Mann-Whitney-U (n_1_ = 60, n_2_ = 35) = 166.5, *p < .001*; BPD-REM vs. HC: Mann-Whitney-U (n_1_ = 60, n_2_ = 35) = 142.0, *p < .001*). These age differences were expected, as the BPD-REM participants constituted former patients with BPD, who were treated in the dialectical behavior therapy inpatient treatment program in Freiburg in the years 1995 through 2002, whereas the patients in the BPD-ACU group were recruited from the waiting list of the residential dialectical behavior therapy treatment program in Mannheim in the years 2008 through 2013. The BPD-REM and the BPD-ACU participants did not differ regarding the percentage of participants in each group taking psychotropic medication. Comorbidities were common in both the patients in the BPD-ACU group as well as the participants in the BPD-REM group. The most frequent co-occurring DSM-IV axis I diagnoses included mood disorders (BPD-ACU: *n* = 38, 63%; BPD-REM: *n* = 9, 25%) and anxiety disorders (BPD-ACU: *n* = 36, 60%; BPD-REM: *n* = 18, 51%) with posttraumatic stress disorder being the most prevalent anxiety disorder in both groups (see Table 1). Nevertheless, patients in the BPD-ACU group had significantly more co-occurring axis I disorders compared to the participants in the BPD-REM group, Mann–Whitney U (n_1_ = 60, n_2_ = 35) = 662.0, *p* < .01.

**Table 1:** Sample characteristics by group

	BPD-ACU (*n* = 60)	BPD-REM (*n* = 35)	HC (n = 60)	*group differences*
Age (in years)				χ^2^(2) = 59.42, *p < .001*
mean (SD)	27.22 (7.01)	43.60 (9.32)	27.83 (6.12)	
median (min – max)	26 (18–45)	41 (31–64)	26 (18–44)	
Psychotropic medication, n (%)	40 (67%)	22 (63%)	–	χ^2^(1) = .437, *p = .51*
Axis I comorbidities, n (%)				
Major depressive disorder	38 (63%)	9 (26%)	–	χ^2^(1) = 12.52, *p ≤ .001*
Dysthymia	3 (5%)	4 (11%)	–	χ^2^(1) = 1.34, *p = .25*
Panic disorder	17 (28%)	5 (14%)	–	χ^2^(1) = 2.45, *p = .12*
Social phobia	10 (17%)	5 (14%)	–	χ^2^(1) = 0.09, *p = .76*
Specific phobia	3 (5%)	4 (11%)	–	χ^2^(1) = 1.34, *p = .25*
Generalized anxiety disorder	4 (7%)	3 (9%)	–	χ^2^(1) = 0.12, *p = .73*
Posttraumatic stress disorder	21 (35%)	12 (34%)	–	χ^2^(1) = 0.01, *p = .94*
Somatization disorder	7 (12%)	0 (0%)		χ^2^(1) = 4.41, *p ≤ .05*
Obsessive-compulsive disorder	6 (10%)	2 (6%)	–	χ^2^(1) = 0.53, *p = .47*
Harmful use of substances	8 (13%)	7 (20%)	–	χ^2^(1) = 0.74, *p = .39*
Bulimia nervosa	10 (17%)	0 (0%)	–	χ^2^(1) = 6.52, *p ≤ .01*
Anorexia nervosa	2 (3%)	1 (3%)	–	χ^2^(1) = 0.02, *p = .90*
Binge eating disorder	7 (12%)	1 (3%)	–	χ^2^(1) = 2.23, *p = .14*
Other eating disorders	4 (7%)	1 (3%)	–	χ^2^(1) = 0.64, *p = .42*
Axis II comorbidities^a^, n (%)				
Cluster A	16 (27%)	0 (0%)	–	χ^2^(1) = 11.66, *p ≤ .001*
Cluster B^b^	6 (10%)	1 (3%)	–	χ^2^(1) = 1.76, *p = .19*
Cluster C	34 (59%)	1 (3%)	–	χ^2^(1) = 28.92, *p ≤ .001*
Number of BPD criteria				U(60,35) = 1.50, *p ≤ .001*
mean (SD)	7.21 (1.35)	1.54 (1.13)	–	
median (min – max)	7 (5–9)	1 (0–4)	–	
Total number of self-reports (maximum of 48)^c^				χ^2^(2) = 1.24, *p = .54*
mean (SD)	42.18 (5.85)	43.74 (6.99)	40.97 (6.99)	
median (min – max)	44 (26–48)	45 (29–48)	43 (24–48)	
Compliance rate^c^, %				χ^2^(2) = 1.25, *p = .54*
mean (SD)	88.78 (12.19)	88.97 (10.73)	91.49 (8.05)	
median (min – max)	91.67 (46–100)	93.75 (60–100)	93.75 (63–100)	
Post-monitoring questionnaire				χ^2^(2) = 17.03, *p ≤ .001*
mean (SD)	2.35 (0.62)	2.10 (0.74)	1.90 (0.60)	
median (min – max)	2.33 (1.17–3.67)	1.83 (1.17–4.00)	1.83 (1.17–4.00)	

Note: ^a^ based on 58 patients in the BPD-ACU group; ^b^ in addition to the BPD diagnosis; ^c^ based on 34 participants in the BPD-REM group; the BPD-ACU and the HC group have been reported in Santangelo et al. [18]

### E-diary assessment and measures

Data on affective instability and instability of self-esteem were collected during participants’ daily lives. After completing the diagnostic assessments, participants were thoroughly instructed and trained regarding the use of the e-diary, which they carried on four consecutive days while undergoing their usual everyday life activities. Participants in the BPD-ACU and the HC group received a palmtop computer (Tungsten-E, Palm Inc., USA) programmed with the IzyBuilder software (IzyData Ltd., Switzerland) to function as an e-diary, whereas the participants in the BPD-REM group received a study smartphone programmed with the movisensXS app (movisens GmbH, Karlsruhe, Germany). We checked for basic differences between the assessment devices and found no differences (cf. [44]).

On the following four days, the e-diary emitted a prompting signal according to a pseudorandomized time-sampling schedule in hourly intervals (60 min ± 10 min) from 10 am to 10 pm. Participants were prompted 12 times a day, resulting in a total of 48 prompts per participant over the four-day assessment period. Each response was automatically time-stamped by the e-diary. After completing four assessment days, participants returned the e-diaries, were debriefed, and financially compensated based on the number of completed data entries (40 to 50 Euros). Moreover, participants were asked about their experiences with the e-diary procedure using a post-monitoring questionnaire with six questions, all on a 5-point rating scale ranging from 1 = not at all to 5 = very much (the questionnaire constitutes an adaption from [45]). Overall, participants reported low to medium reactivity with higher ratings of burdensomeness in the BPD-ACT group (Table 1). Even though the overall test was significant, the differences between the groups were small and the only significant post-hoc difference emerged between the BPD-ACU and the HC groups (Mann-Whitney-U (n_1_ = 60, n_2_ = 60) = 8.1, *p < .001*). The study was approved by the institutional review board of the Medical Faculty Mannheim, Heidelberg University, and all participants provided written informed consent before participating in the study. Participants’ adherence to the e-diary protocol (that is, the number of answered e-diary prompts) was very good with a mean compliance rate of approximately 90% (median = 93.75%) and did not differ between groups (Table 1). However, one of the BPD-REM participants encountered technical problems on the first day of the assessment, and therefore no e-diary data of this person was available. Thus, the BPD-REM e-diary data set comprised data on 34 BPD-REM participants.

At each prompt, participants rated their current affect and self-esteem. To assess participants’ momentary affective states, we used a specifically designed and validated measure for repeated assessments of momentary affective states in e-diary studies [46]. Momentary affective state was conceptualized as varying along two dimensions, and participants rated two bipolar items for each valence (ranging from unpleasant to pleasant) and tense arousal (ranging from restless/under tension to calm/relaxed). In more detail, the item wordings of the valence scale were the German equivalent of “At this moment I feel: unwell–well” and “content–discontent” and of the tense arousal scale “At this moment I feel: agitated–calm” and “relaxed–tense”, whereas the latter item of each scale was reverse coded. Patients with a palmtop computer rated the four bipolar items regarding their momentary affective state on a 7-point rating scale ranging from 0 to 6, whereas those with a study smartphone rated each item on a visual analog scale ranging from 0 to 100. To yield comparable values, ratings of the visual analog scale (0–100) were converted into the 7-point rating scale (0–6) for the four items.

To assess participants’ current self-esteem, we used a four-item short form of the Rosenberg Self-Esteem Scale [47]. Items 1, 2, 9, and 10 of the original scale were adapted to assess the participants’ current status (i.e. the wording “*on the whole, … “* was replaced by “*at the moment, …*”). The item wordings of the items used were “At the moment:” (1) “I am satisfied with myself”; (2) “I think I am no good at all”; (3) “I am inclined to feel that I am a failure”; (4) “I take a positive attitude toward myself”, with items 2 and 3 being reverse coded. The original four-point rating scale was expanded to increase the potential variability in the ratings (see [13, 48]). In more detail, patients with a palmtop computer rated the four items on a 10-point rating scale ranging from 0 to 9, whereas those with a study smartphone rated each item on a visual analog scale ranging from 0 to 100. To yield comparable values, ratings of the visual analog scale (0–100) were converted into the 10-point rating scale (0–9). The items to assess participants’ momentary affective states and self-esteem have been successfully used in prior studies [18, 44]. In the present sample, we conducted variance component analyses to examine whether our measures of valence, tense arousal, and self-esteem were able to assess within-person change over time reliably. The reliability of the items was very good in our sample (valence R_C_ = .76, tense arousal R_C_ = .73, self-esteem R_C_ = .84), which is in line with the high reliability of the e-diary scales reported in our prior studies (see [18, 44]).

### Single point-in-time assessment of the general level of functioning and quality of life

To assess the general level of functioning, we used the interviewer ratings on the Global Assessment of Functioning scale of the DSM-IV (GAF [49]). The GAF scale assesses how severe a person’s mental illness is and how much a person’s symptoms affect his or her everyday life. Interviewers subjectively rate the social, occupational, and psychological functioning of an individual, covering the range from positive mental health to severe psychopathology. The GAF is constructed as an overall measure with 100 scoring possibilities of the level of functioning (1–100), whereas higher scores indicate greater levels of functioning.

Participants in the BPD-REM group filled the World Health Organization Quality of Life questionnaire (WHOQOL-BREF [50]). The WHOQOL-BREF comprises 26 items, which measure four broad domains of quality of life, namely, physical health, psychological health, social relationships, and environment. In addition, there are two items that measure the overall quality of life and general health. Participants are asked to rate how much they have experienced the items in the preceding two weeks on a 5-point rating scale ranging from 1 (not at all) to 5 (extremely/completely/always). The raw domain scores were transformed according to guidelines [50], resulting in a mean domain score that is between 4 and 20, whereas higher scores indicate a greater quality of life. The instrument has good to excellent psychometric properties and constitutes both a reliable and valid measure of participants’ quality of life [51]. In our sample, Cronbach’s alpha for the WHOQOL-BREF was very good (α = .89) with moderate to good Cronbach’s alphas for the four subscales (α_physical health_ = .71, α_psychological health_ = .82, α_social relationships_ = .54, and α_environment_ = .76).

### Data preprocessing and statistical analyses

#### Data preprocessing

We created composite valence, tense arousal, and self-esteem scores by inverse scoring the negatively poled items and then calculating the mean values of the respective items for each administration of the scale. For the variables included in the analyses, possible values ranged from 0 to 6 for valence and tense arousal, and from 0 to 9 for self-esteem.

#### Analyses of instability

In the current study, we applied identical statistical procedures as in the original study [18]. Thus, we calculated three instability indices that allow for examining group differences while taking into account the temporal structure of the unstable processes: Squared successive differences (SSD [52]), probability of acute change (PAC [53]), and aggregated point-by-point changes (APPC [18]), i.e. decreases and increases in relation to the preceding rating. We calculated the SSD by first determining the differences of two consecutive assessments and then squaring these differences. Thus, large differences between two measures are given a higher weightage than smaller differences. We determined the PAC by defining *acute changes*, i.e. the changes in the top 10 percentile of the distribution of successive differences over all persons*.* The cut points corresponding to the 90th percentiles were 2.75 for self-esteem, 2 for valence, and 2.5 for tense arousal. Hence, successive differences were declared acute changes when the differences of two consecutive assessments were equal or greater than these predetermined cut points. To analyze group differences, specific multilevel models were used for analyzing SSD (a gamma model with a log link) and PAC (a logistic model with a logit link) in a two-level model. To examine group differences regarding global instability (i.e. SSD) and the likelihood of extreme changes (i.e. PAC), we analyzed a total of six models, i.e. one model each for SSD and PAC of valence, tense arousal, and self-esteem.

We calculated the APPC by decomposing the self-esteem and valence time series into decreases and increases in relation to the preceding rating of the decreases or increases (i.e. point-by-point changes). Thus, APPC descriptively describe whether specific patterns of increases or decreases characterize instability, i.e. whether changes (ups or downs) are related to specific states (e.g. only during high self-esteem or highly positive valence). By disentangling the time series and decomposing them into point-by-point changes, we obtained multiple decreases and increases in self-esteem and valence for each participant. We aggregated these changes by their momentary starting state into five nearly equal self-esteem bins and valence bins, respectively. For self-esteem decreases the five bins correspond to the following ratings: low = 0.25–2, mid-low = 2.25–3.75, mid = 4–5.5, mid-high = 5.75–7.25, and high self-esteem = 7.5–9, whereas for self-esteem increases the five bins correspond to the ratings: low = 0–1.75, mid-low = 2–3.5, mid = 3.75–5.25, mid-high = 5.5–7, and high self-esteem = 7.25–8.75. For valence decreases the five bins correspond to the ratings: low = 0.5–1.5, mid-low = 2–3, mid = 3.5–4, mid-high = 4.5–5, and high valence = 5.5–6, and for valence increases the five bins correspond to the ratings: low = 0–0.5, mid-low = 1–1.5, mid = 2–2.5, mid-high = 3–4, and high valence = 4.5–5.5. We conducted multilevel analyses to analyze the aggregated between-group changes among these bins (see [18, 20]). To counteract the problem of multiple comparisons, we used the Bonferroni-Holmes correction [54].

We also examined the strength of the association of self-esteem instability with affective instability by analyzing random slope two-level gamma log link models, in which SSD of self-esteem were predicted by SSD of valence and SSD of tense arousal, respectively, at Level 1 in slopes-as-outcomes linear mixed models. That is, we extracted one slope parameter per person, reflecting the association between changes in self-esteem and changes in valence (tense arousal, respectively) of each person. We then used the extracted slopes of these models in linear regression models to predict the single point-in-time assessments of (i) the general level of functioning, i.e. the GAF score; and (ii) the overall quality of life and general health as well as the four domains of quality of life assessed by the WHOQOL-BREF, i.e. physical health, psychological health, social relationships, and environment, in the BPD-REM group. To put it simply, we were interested in whether a strong link between concurrent changes of self-esteem and affect is associated with higher impairments in functioning and quality of life.

We used the R [55] function for generalized linear mixed models “glmer” (package “lme4” [56]) to test our hypotheses. The specific models are described in more detail in Santangelo et al. [18]. We solely report the comparisons of the BPD-REM group with the BPD-ACU and the HC group since the comparisons of the latter two groups have been reported elsewhere [18].

## Results

### Group differences of self-esteem instability and affective instability between BPD-REM and BPD-ACU

The multilevel analyses of the SSD and the PAC of self-esteem instability revealed significant differences between the participants in the BPD-REM group and those in the BPD-ACU group. Specifically, the multilevel SSD analyses, which capture general instability, indicate that participants in the BPD-REM group showed significantly lower instability in self-esteem compared to those in the BPD-ACU group (SSD: β = 0.43, SE = 0.12, z(5690) = 3.53, *p < .001*). The estimated means for the SSD of self-esteem were approximately 54% higher in the BPD-ACU group compared to the BPD-REM participants (4.76 in the BPD-ACU vs. 3.09 in the BPD-REM).

**Fig. 1:**
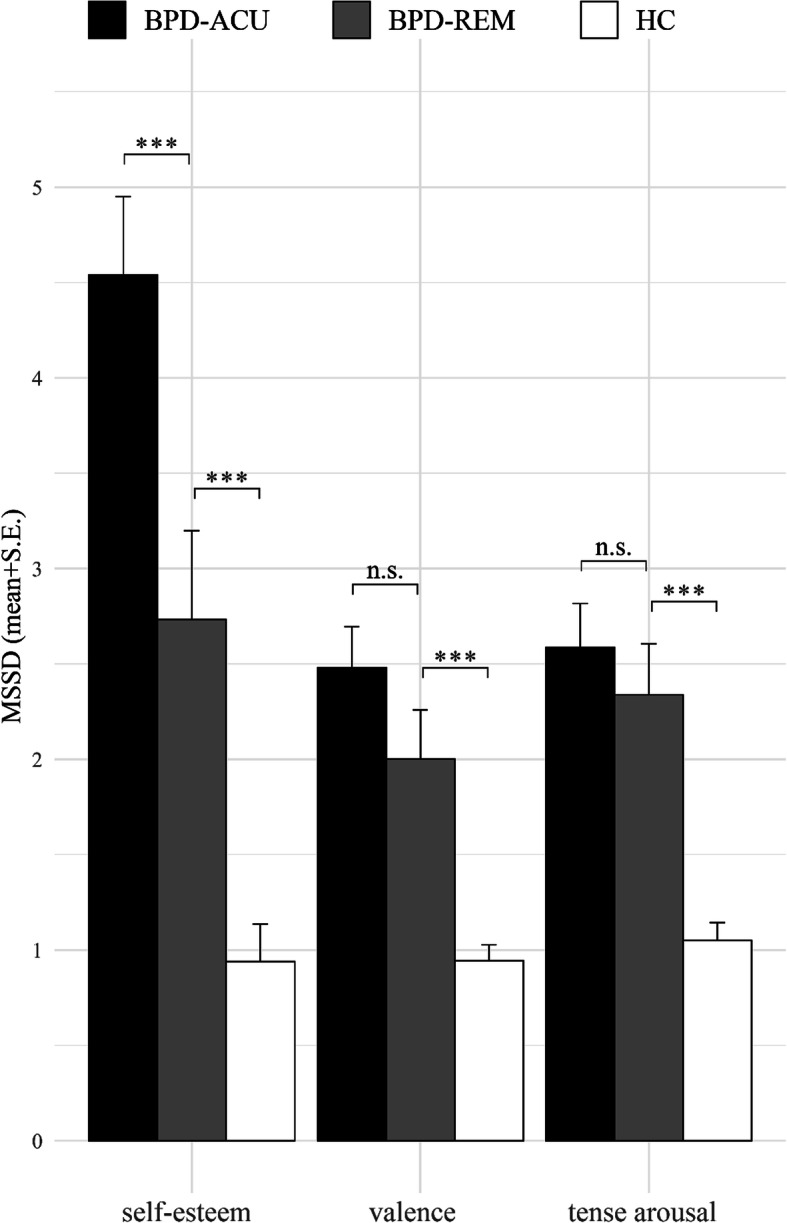
Barplots of the mean squared successive difference (MSSD) per group. Note: MSSD = Average of the squared differences between successive assessments, separate for the patients with acute BPD (BPD-ACU), those who remitted from BPD (BPD-REM), and the healthy controls (HC). *** *p* < .001, ** *p* < .01, * *p* < .05, n.s. = no significant difference (*p* > .05). Significance levels of group differences represent the results of the multilevel models reported in the text. We only report comparisons of the BPD-REM group with the BPD-ACU and the HC group, since the comparison of the BPD-ACU and the HC groups has been reported elsewhere (see Santangelo et al. [18])

In contrast, the two groups did not differ regarding affective instability since the multilevel analyses of the SSD and the PAC of affective instability revealed no significant differences between the participants in the BPD-REM group and the patients in the BPD-ACU group, except for the PAC of valence. Specifically, the multilevel SSD analyses indicate that participants in the BPD-REM group did not show significantly differing instability in valence (SSD: β = 0.15, SE = 0.09, z(5695) = 1.67, *p = .10*) or tense arousal, (SSD: β = 0.05, SE = 0.09, z(5695) = 0.60, *p = .55*) compared to those in the BPD-ACU group. The estimated means for the SSD of valence and tense arousal were only marginally higher in the BPD-ACU patients compared to the BPD-REM participants, namely 3.16 in the BPD-ACU vs. 2.72 in the BPD-REM for valence, and 3.23 in the BPD-ACU vs. 3.06 in the BPD-REM for tense arousal. To illustrate the group differences regarding self-esteem instability and affective instability in a simple way, we calculated the mean SSD per group for self-esteem, valence, and tense arousal (see Fig. 1).

**Fig. 2:**
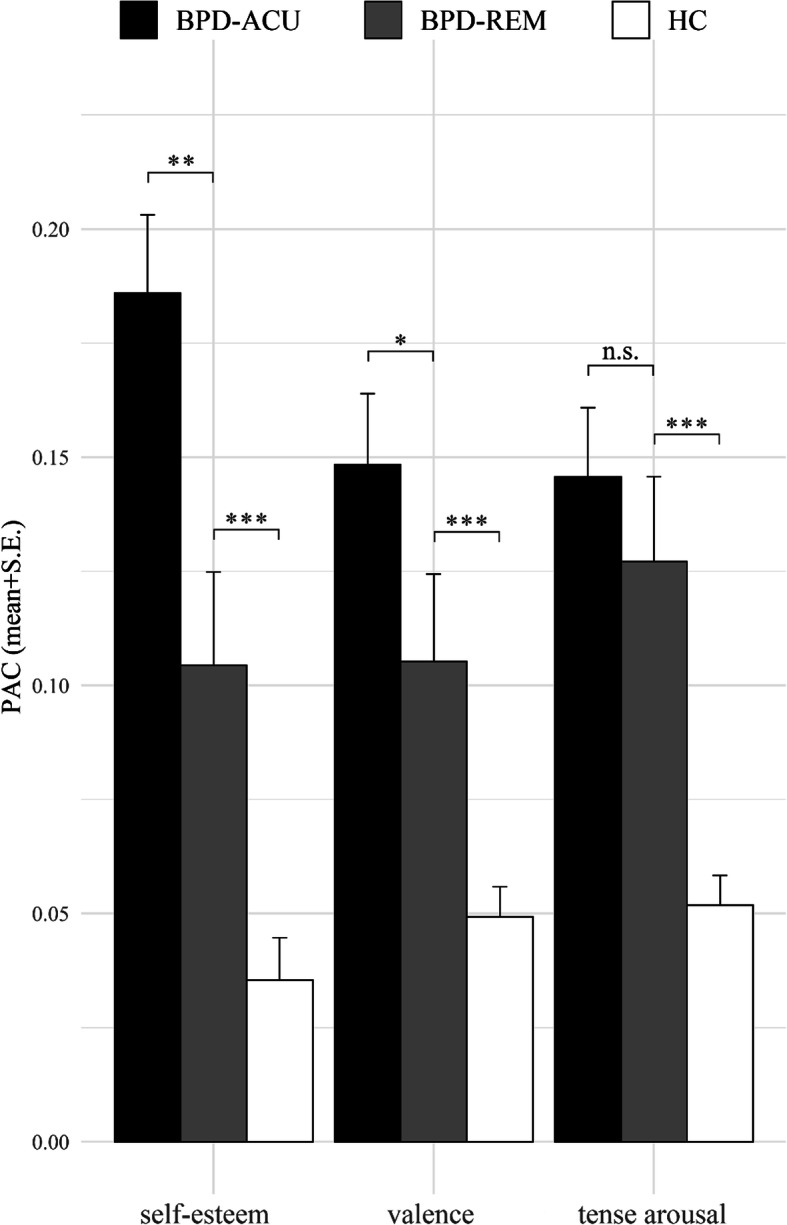
Barplots of the probability of acute change (PAC) per group. Note: PAC = Probability of acute change, i.e. the number of acute changes divided by the total number of changes, separate for the patients with acute BPD (BPD-ACU), those who remitted from BPD (BPD-REM), and the healthy controls (HC). *** *p* < .001, ** *p* < .01, * *p* < .05, n.s. = no significant difference (*p* > .05). Significance levels of group differences represent the results of the multilevel models reported in the text. We only report comparisons of the BPD-REM group with the BPD-ACU and the HC group, since the comparison of the BPD-ACU and the HC groups has been reported elsewhere (see Santangelo et al. [18])

The multilevel PAC analysis of self-esteem is entirely in line with the SSD finding since it showed a significantly heightened probability of occurrences of extreme changes in self-esteem in the BPD-ACU patients compared to the BPD-REM participants (PAC: β = 0.91, SE = 0.31, z(5690) = 2.95, *p < .01*), with the BPD-ACU patients’ risk for an acute change in self-esteem being 2.49 times (95% CI [1.36, 4.57]) higher than the BPD-REM participants’ risk. In contrast to that and in line with the findings regarding the SSD of affect, the multilevel PAC analysis did not reveal significantly heightened occurrences of extreme changes in tense arousal in the BPD-ACU patients compared to the BPD-REM participants (PAC: β = 0.17, SE = 0.24, z(5695) = 0.72, *p = .47*), with the BPD-ACU patients’ risk for an acute change in tense arousal being only marginally higher compared to the BPD-REM participants’ risk, i.e. 1.19 times (95% CI [0.74, 1.91]). However, the multilevel PAC analysis of valence did show a significant difference between BPD-ACU patients and the BPD-REM participants (PAC: β = 0.42, SE = 0.21, z(5695) = 2.07, *p < .05*), with the BPD-ACU patients’ risk for an acute change in valence being 1.53 times (95% CI [1.02, 2.30]) higher than the BPD-REM participants’ risk. Figure 2 depicts the mean PAC per group for self-esteem, valence, and tense arousal.

**Fig. 3:**
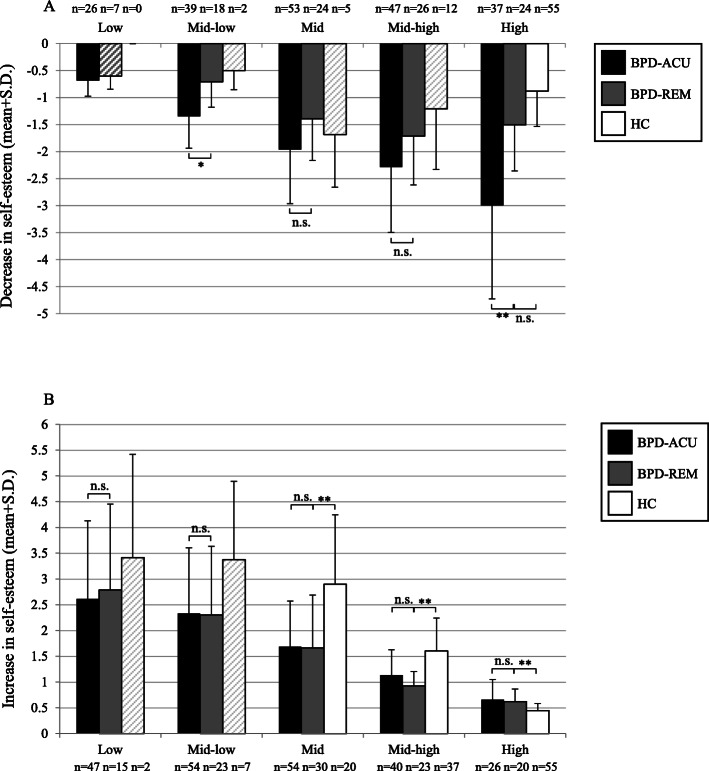
**a**, **b**: Changes in self-esteem in relation to the preceding self-esteem rating across groups. **a** Decreases in self-esteem by bin, i.e. by momentary self-esteem starting state. **b** Increases in self-esteem by bin, i.e. by momentary self-esteem starting state. *** *p* < .001, ** *p* < .01, * *p* < .05, n.s. = no significant difference (*p* > .05); *p*-values are adjusted using the Bonferroni-Holmes correction; crosshatched bars: the small number of participants (< 1/3 of the group) restricts the reliability of the data; thus, the significance tests were not interpreted. We only report comparisons of the BPD-REM group with the BPD-ACU and the HC group, since the comparison of the BPD-ACU and the HC groups have been reported elsewhere [18]

**Fig. 4:**
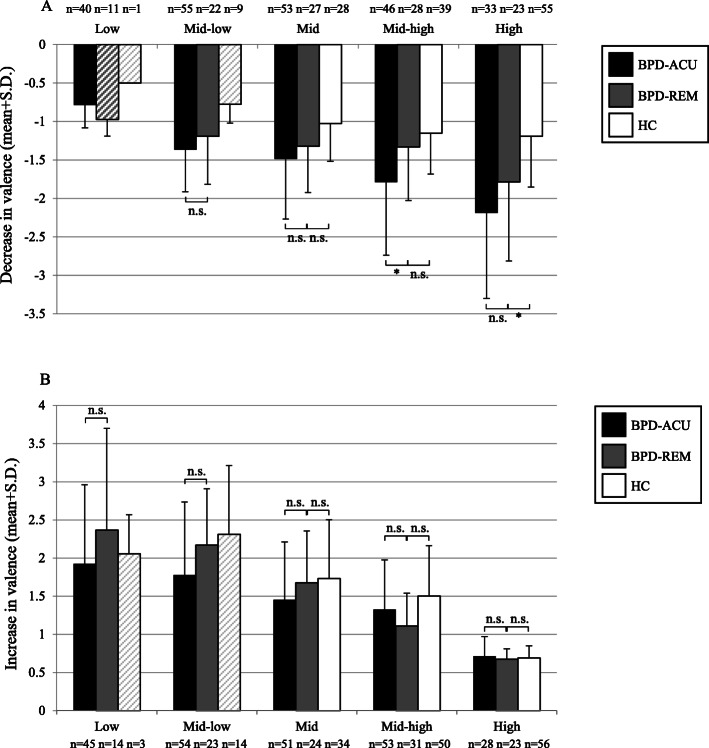
**a**, **b**: Changes in valence in relation to the preceding valence rating across groups. **a** Decreases in valence by bin, i.e. by momentary valence starting state. **b** Increases in valence by bin, i.e. by momentary valence starting state. *** *p* < .001, ** *p* < .01, * *p* < .05, n.s. = no significant difference (*p* > .05); p-values are adjusted using the Bonferroni-Holmes correction; crosshatched bars: the small number of participants (< 1/3 of the group) restricts the reliability of the data; thus, the significance tests were not interpreted. We only report comparisons of the BPD-REM group with the BPD-ACU and the HC group, since the comparison of the BPD-ACU and the HC groups has been reported elsewhere (see Santangelo et al. [18])

An in-depth analysis of the decreases and increases in relation to the preceding rating revealed that patients in the BPD-REM group experienced lower decreases in self-esteem irrespective of the self-esteem bin (i.e. the starting state) in comparison to those in the BPD-ACU group. Figure 3a impressively shows that sudden decreases in patients with BPD-ACU were especially pronounced when in a high self-esteem state (almost two times larger in the BPD-ACU compared to the BPD-REM group). Furthermore, the groups significantly differed in the mid-low bin with higher self-esteem decreases in the BPD-ACU group compared to the BPD-REM group. The sudden drops in low self-esteem states were smaller, most likely due to floor effects. Please note that the data in the low self-esteem bin should be interpreted with caution because only a few BPD-REM participants reported a decrease in this bin; consequently, we did not interpret the statistical test for group differences in the low self-esteem bin. Table 2 provides further information regarding the descriptive statistics of the aggregated point-by-point changes in self-esteem. The upper part of the table shows the mean starting state of each bin, the number of participants, their total number of decreases per bin, and the median, minimum, and maximum number of changes per person in that specific bin for each of the three groups. In addition, the table provides information regarding the magnitude of the decreases that these participants reported. In contrast to the decreases, Fig. 3b, which depicts increases in self-esteem, did not reveal significant differences between the BPD-REM and the BPD-ACU participants irrespective of the self-esteem starting state (see the lower part of Table 2 for the descriptive statistics regarding the increases in self-esteem for the three groups). Thus, the pattern of instability of self-esteem in the BPD-REM group seems to be characterized by lower decreases in self-esteem compared to the BPD-ACU, whereas the group differences are most pronounced in higher self-esteem starting states. However, there are no differences between the BPD-REM group and the BPD-ACU regarding increases in self-esteem. Concerning the decreases in valence, Fig. 4a indicates that the group differences between the BPD-REM and the BPD-ACU are not as pronounced with smaller differences in all bins, even in the mid-high and high valence bins (although the group difference in the mid-high valence bin was significant). Regarding increases in valence, Fig. 4b depicts no significant differences between the BPD-REM and the BPD-ACU groups. Table 3 provides further descriptive statistics of the aggregated point-by-point changes in valence.

**Table 2:** Descriptive statistics of the aggregated point-by-point changes in self-esteem by group: The mean and standard deviation of the starting state, the number of participants, their total number of decreases (increases, respectively) per bin and the median, minimum, and maximum number of changes as well as information regarding the magnitude of the decreases (increases, respectively) that these participants reported, including the mean and standard deviation, median, minimum, and maximum

**Decreases in self-esteem**							
	Bin	Starting state: Mean (Sd)	Number of participants	Total number of decreases	Number of decreases: Median (min – max)	Decreases: Mean (Sd)	Decreases: Median (min – max)
BPD-ACU	Low	1.13 (0.53)	26	100	3 (1–15)	−0.68 (0.29)	−0.63 (− 0.25 – −1.50)
	Mid-low	2.97 (0.52)	39	166	3 (1–15)	−1.35 (0.59)	−1.25 (− 0.50 – −3.00)
	Mid	4.72 (0.47)	53	266	4 (1–15)	− 1.96 (1.00)	− 1.92 (− 0.48 – −5.25)
	Mid-high	6.51 (0.49)	47	217	4 (1–11)	−2.28 (1.21)	− 2.05 (− 0.61 – −5.87)
	High	8.26 (0.54)	37	200	5 (1–15)	− 2.99 (1.73)	− 2.63 (− 0.62 – −7.08)
BPD-REM	Low	1.60 (0.42)	7	13	2 (1–13)	− 0.60 (0.24)	−0.75 (− 0.25 – − 0.87)
	Mid-low	3.23 (0.43)	17	52	1 (1–12)	− 0.70 (0.47)	−0.56 (− 0.25 – − 2.25)
	Mid	4.72 (0.45)	23	102	4 (1–13)	− 1.39 (0.77)	− 1.25 (− 0.31 – − 2.75)
	Mid-high	6.58 (0.48)	25	147	5 (1–15)	− 1.71 (0.90)	−1.43 (− 0.25 – − 3.50)
	High	8.28 (0.52)	24	205	7.5 (1–19)	− 1.50 (0.85)	− 1.25 (− 0.50 – − 3.11)
HC	Low	–	0	0	–	–	–
	Mid-low	2.88 (0.18)	2	2	1 (1–1)	− 0.50 (0.35)	−0.50 (− 0.25 – − 0.75)
	Mid	4.70 (0.61)	3	9	1 (1–3)	− 1.68 (0.97)	−1.25 (− 0.75 – − 3.00)
	Mid-high	6.59 (0.55)	12	41	2 (1–13)	− 1.21 (1.12)	−0.83 (− 0.25 – − 4.12)
	High	8.53 (0.45)	55	485	9 (1–18)	− 0.88 (0.65)	− 0.67 (− 0.25 – − 3.83)
**Increases in self-esteem**							
	Bin	Starting state: Mean (Sd)	Number of participants	Total number of increases	Number of increases: Median (min – max)	Increases: Mean (Sd)	Increases: Median (min – max)
BPD-ACU	Low	0.77 (0.62)	47	256	5 (1–16)	2.61 (1.52)	2.25 (0.25–6.75)
	Mid-low	2.71 (0.49)	54	229	3 (1–12)	2.33 (1.27)	2.25 (0.33–5.25)
	Mid	4.46 (0.48)	54	233	4 (1–11)	1.69 (0.88)	1.52 (0.38–4.25)
	Mid-high	6.21 (0.48)	40	163	3 (1–11)	1.14 (0.49)	1.11 (0.25–2.50)
	High	7.87 (0.46)	26	72	2 (1–14)	0.66 (0.39)	0.52 (0.25–1.50)
BPD-REM	Low	1.23 (0.50)	15	35	1 (1–6)	2.79 (1.67)	2.50 (0.50–5.50)
	Mid-low	2.86 (0.48)	22	103	3.5 (1–14)	2.30 (1.34)	2.23 (0.64–5.25)
	Mid	4.43 (0.47)	29	126	4 (1–12)	1.66 (1.03)	1.44 (0.38–4.25)
	Mid-high	6.28 (0.52)	23	129	5 (1–12)	0.92 (0.89)	0.89 (0.47–1.43)
	High	7.82 (0.45)	20	120	5 (1–13)	0.62 (0.25)	0.62 (0.25–1.17)
HC	Low	1.19 (0.31)	2	4	2 (1–3)	3.42 (2.00)	3.42 (2.00–4.83)
	Mid-low	2.78 (0.47)	7	20	2 (1–7)	3.38 (1.52)	3.75 (1.25–5.50)
	Mid	4.71 (0.41)	20	46	1.5 (1–10)	2.90 (1.35)	3.25 (0.85–4.75)
	Mid-high	6.51 (0.44)	37	126	2 (1–16)	1.61 (0.64)	1.50 (0.38–3.00)
	High	8.16 (0.47)	55	422	8 (1–16)	0.45 (0.13)	0.43 (0.25–1.00)

*BPD-ACU* patients with an acute Borderline personality disorder; *BPD-REM* symptomatically remitted patients with a loss of BPD diagnosis (< 5 diagnostic BPD criteria); *HC* healthy controls

**Table 3:** Descriptive statistics of the aggregated point-by-point changes in valence by group: The mean and standard deviation of the starting state, the number of participants, their total number of decreases (increases, respectively) per bin and the median, minimum, and maximum number of changes as well as information regarding the magnitude of the decreases (increases, respectively) that these participants reported, including the mean and standard deviation, median, minimum, and maximum

**Decreases in valence**							
	Bin	Starting state: Mean (Sd)	Number of participants	Total number of decreases	Number of decreases: Median (min – max)	Decreases: Mean (Sd)	Decreases: Median (min – max)
BPD-ACU	Low	1.16 (0.38)	40	121	2 (1–11)	−0.79 (0.30)	−0.75 (−0.50 – −1.50)
	Mid-low	2.62 (0.40)	55	253	4 (1–12)	−1.37 (0.55)	−1.25 (− 0.50 – −3.00)
	Mid	3.73 (0.25)	53	188	3 (1–8)	−1.49 (0.78)	− 1.30 (− 0.50 – −4.00)
	Mid-high	4.78 (0.25)	46	153	3 (1–9)	− 1.79 (0.95)	−1.79 (− 0.50 – −5.00)
	High	5.81 (0.24)	33	111	2 (1–9)	−2.19 (1.11)	−2.19 (− 0.50 – − 4.50)
BPD-REM	Low	1.27 (0.34)	11	22	1 (1–5)	−0.97 (0.21)	−1.00 (− 0.67 – − 1.50)
	Mid-low	2.69 (0.39)	22	78	3 (1–13)	− 1.19 (0.63)	− 1.00 (− 0.50 – − 2.50)
	Mid	3.85 (0.23)	27	112	4 (1–13)	−1.32 (0.60)	− 1.17 (− 0.50 – − 2.50)
	Mid-high	4.79 (0.25)	28	154	5 (1–12)	−1.33 (0.69)	− 1.11 (− 0.50 – − 3.37)
	High	5.75 (0.25)	23	113	4 (1–11)	−1.78 (1.03)	− 1.60 (− 0.50 – − 5.00)
HC	Low	1.00 (0.00)	1	2	2 (2–2)	− 0.50 (−-)	− 0.50 (− 0.50 – − 0.50)
	Mid-low	2.70 (0.37)	9	27	2 (1–7)	−0.78 (0.25)	− 0.75 (− 0.50 – − 1.08)
	Mid	3.76 (0.25)	28	78	2 (1–7)	−1.03 (0.49)	− 1.00 (− 0.50 – − 2.50)
	Mid-high	4.84 (0.23)	39	176	4 (1–11)	−1.15 (0.53)	− 1.00 (− 0.50 – − 3.00)
	High	5.80 (0.23)	55	295	5 (1–14)	− 1.19 (0.66)	−1.00 (− 0.50 – − 4.00)
**Increases in valence**							
	Bin	Starting state: Mean (Sd)	Number of participants	Total number of increases	Number of increases: Median (min – max)	Increases: Mean (Sd)	Increases: Median (min – max)
BPD-ACU	Low	0.21 (0.25)	45	180	4 (1–13)	1.92 (1.04)	1.75 (0.50–4.50)
	Mid-low	1.25 (0.25)	54	183	3 (1–7)	1.78 (0.96)	1.50 (0.50–5.00)
	Mid	2.23 (0.25)	51	184	4 (1–8)	1.45 (0.76)	1.25 (0.50–3.50)
	Mid-high	3.33 (0.40)	53	205	3 (1–12)	1.32 (0.65)	1.25 (0.50–3.00)
	High	4.90 (0.41)	28	82	2 (1–8)	0.71 (0.26)	0.63 (0.50–1.50)
BPD-REM	Low	0.35 (0.25)	14	30	2 (1–5)	2.37 (1.34)	2.29 (0.50–5.00)
	Mid-low	1.25 (0.25)	23	58	2 (1–9)	2.17 (0.74)	2.17 (0.86–3.50)
	Mid	2.25 (0.25)	24	78	3 (1–8)	1.67 (0.68)	1.75 (0.63–3.25)
	Mid-high	3.56 (0.42)	31	181	6 (1–12)	1.11 (0.43)	1.06 (0.50–2.50)
	High	4.86 (0.37)	23	106	4 (1–10)	0.67 (0.14)	0.67 (0.50–0.92)
HC	Low	0.50 (0.00)	3	5	1 (1–3)	2.06 (0.51)	2.17 (1.50–2.50)
	Mid-low	1.33 (0.24)	14	23	1 (1–3)	1.31 (0.90)	2.00 (1.00–3.50)
	Mid	2.29 (0.25)	34	76	1.5 (1–8)	1.73 (0.77)	1.50 (0.50–4.00)
	Mid-high	3.53 (0.43)	50	233	5 (1–11)	1.50 (0.67)	1.33 (0.50–3.00)
	High	4.90 (0.40)	56	317	5 (1–14)	0.69 (0.67)	0.67 (0.50–1.08)

*BPD-ACU* patients with an acute Borderline personality disorder; *BPD-REM* symptomatically remitted patients with a loss of BPD diagnosis (< 5 diagnostic BPD criteria); *HC* healthy controls

### Group differences of self-esteem instability and affective instability between BPD-REM and HC

Participants in the BPD-REM group exhibited heightened instability of self-esteem and affective instability compared to the HC. The group differences are depicted in Figs. 1 and 2. The multilevel SSD analyses revealed that, compared to participants in the HC group, those in the BPD-REM group showed significantly higher instability in self-esteem (SSD: β = − 0.61, SE = 0.12, z(5690) = − 5.05, *p < .001*) and affect, both valence (SSD: β = − 0.37, SE = 0.09, z(5695) = − 4.20, *p < .001*) and tense arousal (SSD: β = − 0.44, SE = 0.09, z(5695) = − 4.94, *p < .001*). The group differences are further delineated in the comparison of the estimated means, as the estimated means for the SSD of self-esteem were almost two times (1.85) higher in the BPD-REM group compared to the HC group, and the estimated means for the SSD of valence and tense arousal were approximately one and a half times (1.45 for valence; 1.55 for tense arousal) higher. The multilevel PAC analyses are completely in line with these findings showing significantly elevated occurrences of extreme changes in self-esteem (PAC: β = − 1.46, SE = 0.34, z(5690) = − 4.26, *p < .001*), in valence (PAC: β = − 0.74, SE = 0.21, z(5695) = − 3.44, *p < .001*), and in tense arousal (PAC: β = − 0.99, SE = 0.25, z(5695) = − 3.89, *p < .001*) in the BPD-REM participants compared to the HC.

An in-depth analysis of the decreases and increases in relation to the preceding rating revealed that participants in the BPD-REM group experienced higher decreases in self-esteem regardless of the self-esteem bin (with the only exception being those in the mid self-esteem bin) in comparison to those in the HC group (Fig. 3a). However, the differences between the BPD-REM participants and those in the HC group were not as evident as those in the BPD-ACU group. Please note that the data in the lower through mid-high self-esteem bins should be interpreted with caution because only a few HC participants reported decreases in these bins; consequently, we did not interpret the statistical tests for group differences in these bins. Figure 3b, which depicts increases or repairs in self-esteem, depicts significant differences between the BPD-REM and the HC participants, whereas the increases in the HC participants are generally larger independent of the self-esteem starting state (from the low through mid-high self-esteem bin). In brief, the pattern of instability in the BPD-REM group seems to be characterized by larger decreases in self-esteem irrespective of the self-esteem starting states and by lower increases in self-esteem compared to the HC participants. Thus, even after a loss of BPD diagnosis, the BPD-REM participants seem to suffer from larger drops in self-esteem and a longer time needed to recover from such sudden drops compared to the HC. With regard to the decreases and increases in valence, Fig. 4a and b display that the group differences between the BPD-REM and the HC were marginal with only small and non-significant differences across most bins. However, Fig. 4a indicates that sudden decreases were significantly higher in the BPD-REM group compared to the HC group when in a positive emotional state, i.e. in the high valence bin.

### Impact of the association between affective changes and changes in self-esteem on functioning and quality of life in BPD-REM

The linear regression models revealed that neither the strength of the association between SSD of valence and SSD of self-esteem nor that between SSD of tense arousal and SSD of self-esteem significantly predicted the level of functioning, i.e. the GAF score, in the BPD-REM group (see Table 4). Similarly, neither of the two were significantly predictive of the overall quality of life and general health self-reports in the WHOQOL-BREF (slope valence: F(1, 31) = 0.54, *p = .47*; slope tense arousal: F(1, 31) = 0.73, *p = .40*) nor the four domains of quality of life assessed by the WHOQOL-BREF (Table 4). Yet, the slope between SSD of tense arousal on SSD of self-esteem indicate an impact on the domains of psychological health as well as social relationships, since a higher slope, i.e. a higher association between changes in tense arousal and changes in self-esteem seem to be associated with a lower quality of life in these two domains, even though in both cases the predictor missed the level of significance (*p < .06* and *p < .07*, see Table 4). These results suggest that the association between changes in tense arousal and changes in self-esteem have a potential effect on the domains of psychological health and social relationships of quality of life in the participants in the BPD-REM group.

**Table 4:** Slopes between SSD of valence and SSD of self-esteem as well as those between SSD of tense arousal and SSD of self-esteem predicting the level of functioning (GAF) and different domains of quality of life (based on WHOQOL-BREF) in the BPD-REM participants

Dependent variable	Independent variable	Estimate	Df	F	*p*
*Outcome: Level of functioning*					
GAF	slp valence	1.49	1, 32	0.01	*.91*
GAF	slp tense arousal	18.45	1, 32	0.56	*.46*
*Outcome: Quality of life*					
Physical health	slp valence	−0.52	1, 32	0.03	*.86*
Physical health	slp tense arousal	3.07	1, 32	0.33	*.57*
Psychological health	slp valence	−3.24	1, 32	1.21	*.28*
Psychological health	slp tense arousal	−10.45	1, 32	3.82	*.06*
Social relationships	slp valence	−4.13	1, 32	1.56	*.22*
Social relationships	slp tense arousal	−11.23	1, 32	3.45	*.07*
Environment	slp valence	0.20	1, 32	0.01	*.94*
Environment	slp tense arousal	1.39	1, 32	0.08	*.78*

*GAF* Global assessment of functioning scale of the DSM-IV. Range of possible values = 1–100, whereas higher scores indicate greater levels of functioning; *WHOQOL-BREF* World Health Organization quality of life questionnaire, 26 items version, which assesses the overall quality of life and general health as well as four domains of quality of life, i.e. physical health, psychological health, social relationships, and environment. Range of possible values = 4–20, whereas higher scores indicate a greater quality of life; *slp valence* slope SSD of valence on SSD of self-esteem; *slp tense arousal* slope SSD of tense arousal on SSD of self-esteem

## Discussion

In the present study, we sought to investigate further the instability in self-esteem and affective instability in everyday life in BPD. We compared a sample of remitted BPD participants, i.e. patients with a loss of diagnosis at the time of the e-diary assessment, with acute patients with BPD and HC participants utilizing e-diaries, high-frequency sampling, and various time-sensitive instability indices. We found significantly lower self-esteem instability in the BPD-REM participants compared to the BPD-ACU patients, whereas mainly no significant differences regarding affective instability emerged. On the other hand, the BPD-REM participants consistently showed significantly heightened instability in both self-esteem as well as affect compared to the HC participants. We furthermore addressed potential associations between the strength of the association between changes in affect and changes in self-esteem and the level of functioning as well as the quality of life in the BPD-REM participants. Even though the analyses did not reveal significant associations with the level of functioning nor the quality of life, our results indicate a potential effect of the strength of the intertwinement of changes in tense arousal and self-esteem on the quality of life domains of psychological health and social relationships in the BPD-REM participants. Peculiarly, a greater strength between changes in tense arousal and changes in self-esteem indicated a lower quality of life in these two domains. Taken together, our results suggest that self-esteem instability is lower in remitted BPD, whereas the levels of affective instability are mainly comparable in acute BPD and remitted BPD. However, BPD-REM participants still show heightened self-esteem instability and affective instability in comparison to HC. The strength between changes in tense arousal and changes in self-esteem tentatively hint to a lower quality of life in the domains of psychological health and social relationships in the BPD-REM participants.

Examining the instability of self-esteem seems a promising avenue for future research since our cross-sectional results indicate that it declines after remission from BPD, whereas the affective instability seems to persist. Previous e-diary studies mostly addressed affective instability in BPD (for a review see [5]). However, those studies including clinical control groups in order to address the specificity of affective dysregulation for BPD, largely failed, e.g. using various instability indices [20], subcomponents of affective dysregulation [21], emotional granularity [22], or emotion sequences [23]. Therefore, it has been suggested that affective instability constitutes a transdiagnostic marker of affective dysregulation. In our study, BPD-REM participants were significantly older than BPD-ACU and HC participants, and it has been discussed whether affective instability declines with older age. A recent comprehensive cross-sectional e-diary study from our group found that global affective instability (i.e. SSD) in acute BPD patients’ everyday lives indeed declined with years of age [44]. However, in the current study, we found no differences regarding global affective instability between the, on average younger, acute patients in the BPD-ACU group and the, on average older, remitted participants in the BPD-REM group. In addition, a cross-sectional study [57] analyzing the intraindividual standard deviation of end-of-day self-esteem ratings over 25 days showed that, in a large sample of healthy subjects, participants’ self-esteems tended to become more stable with older age (participants age ranged from 13 to 72 years, whereas most participants were in the range from age 20 to 39 years). Since this study has some important methodological differences (daily assessments, intraindividual standard deviation, healthy sample), we analyzed the association between self-esteem instability and years of age in our sample and found no significant association in none of the three groups (BPD-ACU: β = − 0.01, SE = 0.02, Z = − 3.89, *p = .68*; BPD-REM: β = 0.01, SE = 0.02, Z = 0.61, *p = .54*; HC: β = 0.02, SE = 0.03, Z = 0.64, *p = .52*). Moreover, we examined the possibility of a cohort effect, i.e. familiarity with technology, that may have influenced the results in our sample with diverging mean years of age in the three groups. Directly addressing the association between ratings of the discomfort of the e-diary assessments (assessed by the post-monitoring questionnaire) and years of age revealed no association in our sample (rho = −.05, *p* = .43). Moreover, the age range within the three groups was large (around 30 years, see Table 1) and thus, there was variability in the years of age in all three groups and the age range within each group was greater than the age differences between the groups. Thus, age differences in the BPD-ACU and the BPD-REM groups cannot explain our findings of significantly heightened instability of self-esteem but mainly similar affective instability in the BPD-REM and the BPD-ACU groups.

Furthermore, our finding of comparable levels of affective instability in participants with remitted BPD and patients with acute BPD is in line with results from prospective multi-wave follow-up studies of BPD. Using retrospective self-report measures to assess psychopathology, such as interviews and questionnaires, these studies suggest differential levels of stability across BPD features with the criterion of affective instability persisting over a longer time [31] and being most prevalent over the follow-ups with higher shares of patients still fulfilling the criterion compared to other criteria such as unstable sense of self (e.g. [28, 30]). Even though these studies examining the course of individual symptoms of BPD demonstrated an overall decrease in all symptoms, results indicate that the diagnostic criterion of unstable sense of self remits more frequently and at a quicker rate than the criterion of affective instability. The criterion of an unstable sense of self in everyday life has been neglected in e-diary studies until lately. However, recent e-diary studies brought new attention to the importance of self-esteem in BPD showing heightened instability in self-esteem in daily life in BPD compared to HC [18], as well as highlighting its associations with engaging in dysfunctional behaviors [19]. In the study at hand, we cross-sectionally compared acute vs. remitted BPD patients, and no clinical control group was included in the study. Thus, no statement can be made regarding whether our findings of heightened self-esteem instability are BPD-specific or whether they constitute a transdiagnostic phenomenon. A multitude of prior e-diary studies indicates that affective instability constitutes a transdiagnostic marker of dysregulation in the affective system. For a better understanding of the specificity, as a first step, it is of high relevance to examine whether self-esteem instability and affective instability differ between individuals with a current BPD disorder, remitted disorder, and non-clinical controls, i.e. whether they are indicative of clinical group belonging. Studies including clinical controls are clearly warranted to proceed to the next stage in order to examine further whether instability of self-esteem is specific for BPD.

To examine the assumption that affective changes that are accompanied by changes in self-esteem are experienced as more burdensome and threatening, we tested whether the strength of the association between changes of valence and self-esteem and that between changes of tense arousal and self-esteem predict the level of functioning and the quality of life in the BPD-REM participants. We found no association with the level of functioning, i.e. the GAF score, and the strength of the associations between changes in affect and self-esteem. Though the GAF’s advantage of simplicity [58], it comes with a number of limitations. Most importantly, the reliability of the scale tends to be low and not sufficient in the routine clinical setting [59], as is the validity [60], especially the predictive validity [61]. Moreover, studies found that the rating score can be influenced by raters’ attitude towards the GAF and their knowledge of the patients’ day-to-day life, among other confounding variables [61, 62]. Consequently, the GAF was excluded from the DSM-5 [1].

The associations between changes in affect and changes in self-esteem were not significant predictors of the self-reported overall quality of life or general health. However, even though not significant, results indicate that the association between changes in tense arousal and changes in self-esteem have a potential effect on the domains of psychological health and social relationships of quality of life in the participants in the BPD-REM group. Most likely, these effects are of smaller magnitude, and our study of 35 participants in the BPD-REM group was insufficiently powered to reveal smaller effects. Nonetheless, we would have expected to find an effect of the associations in the two domains of the quality of life questionnaire, in which we found marginal significant effects, i.e. psychological health and social relationships, since large-scale follow-up studies revealed that psychosocial functioning often remains impaired and only a few patients attain social and vocational competence after symptomatic remission (e.g [28, 33, 34]). Future studies should further examine the effects of the associations between changes in affect that are accompanied by changes in self-esteem on participants’ well-being and quality of life since they have great clinical significance.

Several limitations of the current study deserve mention. First, our study is limited in that it is based on cross-sectional data. Only longitudinal studies can definitely speak to the trajectories of affective instability and self-esteem instability over the course of BPD and after remission from it, i.e. the loss of diagnosis. However, there are no longitudinal e-diary studies at hand, and our study is the first that compares affective instability and self-esteem instability in acute and remitted BPD. Moreover, attrition rates can bias the results of longitudinal studies through the loss of participants during follow-up, often due to behaviors strongly associated with the disorder itself. To ensure basic comparability between groups in our cross-sectional study, we assessed everyday life symptomatology in patients currently waiting for inpatient treatment on our specialized BPD treatment unit, as well as former patients who underwent this residential treatment on our specialized unit several years ago. Our main findings that self-esteem instability is lower after remission from BPD, whereas affective instability is still heightened and comparable to that of acute BPD patients, are in accordance with prospective multi-wave studies in BPD using retrospective interviews and questionnaires [28, 31]. These findings collectively suggest that the instability of self-esteem seems to remit faster, whereas the affective instability seems to persist for a longer time, even after remission from BPD. This consistency indicates that the findings observed in this study cannot be attributed solely to artifacts such as longer duration of illness, the time elapsed since the loss of diagnosis, or selective mortality. Nonetheless, the results of this study should be replicated in a longitudinal e-diary study before strong conclusions are drawn.

Second, given that only female participants were included in our study, the generalizability of the findings is limited, and the results may not be valid for male patients with BPD. However, the use of an entirely female sample also reduced the heterogeneity of the sample, which may have been useful, given the literature on sex differences in affect [63] and self-esteem [64]. The results of this study should be replicated in a mixed-sex sample including male patients with BPD.

Third, we defined symptomatic remission as a loss of BPD diagnosis. Patients who had previously met ≥5 diagnostic criteria for BPD but dropped below the diagnostic threshold on the continuum of BPD criteria and fulfilled less than five BPD diagnostic criteria within the past year at the time of the e-diary assessment were considered remitted. Thus, our definition of remission is rather liberal. However, since only three BPD-REM participants fulfilled four diagnostic criteria for BPD, a more conservative definition of remission considering only participants fulfilling ≤3 diagnostic criteria for BPD did not change our findings regarding instability in self-esteem and affective instability (results available upon request). Moreover, the definitions of symptomatic remission from BPD show substantial variation in previous studies, and no consensus has been reached.

Fourth, the patients in the BPD-ACU group were diagnosed with a variety of co-occuring axis I and axis II disorders. We were unable to address the influence of different comorbid diagnoses on self-esteem instability and affective instability due to our restricted sample size. Thus, no statement can be made regarding whether our findings are independent of any comorbidity. Comorbidity is the rule rather than the exception in BPD [65]. Therefore, a sample of BPD patients with high comorbidity rates constitutes a representative, non-artificial sample. In contrast, BPD patients without comorbid disorders cannot be seen as representative of the BPD population [66]. Additionally, no clinical control group was included in the study. Thus, we cannot make any statement whether our findings of heightened self-esteem instability in acute BPD are BPD-specific or whether they constitute a transdiagnostic phenomenon (as does affective instability). Studies including clinical controls are clearly needed to assess whether self-esteem instability is specific to acute BPD or associated with underlying psychopathology other than BPD.

Fifth, we did not consider emotionally or self-esteem relevant events or triggers (e.g. interpersonal events) that might have influenced participants’ ratings during the e-diary assessment period. Because events or triggers might differ between groups and given the growing recognition of the importance of contextual factors in e-diary studies (e.g. [67]), assessments of relevant events should be included and examined in future studies.

Despite these limitations, the current study significantly deepens our understanding of the unstable psychological processes involved in BPD. By extending prior e-diary research on self-esteem instability and affective instability in BPD, this study conducted in remitted BPD participants’ everyday lives builds on growing evidence for the importance of self-esteem instability. Our findings have several clinical implications and provide interesting avenues for consecutive research. E-diaries provide the possibility of in-vivo diagnostic assessments of the severity and time-dependency of dynamic symptoms in daily life and, thus, can help to support clinical diagnoses and decision making. Repeated assessments in participants’ daily lives seem especially fruitful for the evaluation of therapeutic interventions such as dialectical behavior therapy [68], which targets these domains of instability. E-diaries present a promising tool for clinical research by tracking potential changes in self-esteem and affective instability by therapeutic interventions over the course of the psychotherapeutic therapy in the most relevant contexts of all, patients’ everyday lives.

## Conclusion

In conclusion, this study builds on growing evidence for the importance of self-esteem instability in BPD. For a better understanding of the specificity, as a first step, it is highly interesting to examine whether self-esteem instability and affective instability are indicative of clinical group belonging, i.e. whether they differ between individuals with a current BPD disorder, remitted disorder, and non-clinical controls. We used e-diaries with high-frequency sampling and time-sensitive instability indices to examine the instability of self-esteem and affective instability in remitted BPD participants. Our results indicate lower self-esteem instability in remitted BPD participants compared to acute patients with BPD, whereas affective instability is comparably elevated in both groups, and no significant differences regarding affective instability were apparent between the two groups. In comparison to participants in the HC group, those with remitted BPD still showed significantly higher instability, both regarding self-esteem and affect. Moreover, the results of subsequent analyses indicate that the strength between affective changes and changes in self-esteem has a potential impact on the quality of life domains of psychological health and social relationships in the BPD-REM participants. However, these results are tentative and warrant replication in a larger sample of participants after remission from BPD. Further research on self-esteem instability seems a promising avenue to gain further insight into the specificity of the symptom and its associations with psychopathology.

## Data Availability

We are unable to share any data publicly because we used a form of informed consent in which we assert participants to share the data only with researchers of our research lab and associated researchers. We did not explicitly ask whether participants agree to make their anonymized data available online. Thus, sharing participants’ data would violate confidentiality.

## Abbreviations

APPC: Aggregated point-by-point changes
BPD: Borderline personality disorder
BPD-ACU: Patients with an acute borderline personality disorder
BPD-REM: Symptomatically remitted patients with a loss of BPD diagnosis (i.e. fulfilling < 5 diagnostic BPD criteria)
CI: Confidence interval
DSM-IV/DSM-5: American Psychiatric Association’s diagnostic and statistical manual of mental disorders, 4th edition and 5th edition, respectively
GAF: Global assessment of functioning scale of the DSM-IV
HC: Healthy controls
IPDE: International personality disorder examination
PAC: Probability of acute change
SCID–I: Structured clinical interview for DSM–IV axis I disorders
SCID–II: Structured clinical interview for DSM–IV axis II disorders
slp valence: slope SSD of valence on SSD of self-esteem
slp tense arousal: slope SSD of tense arousal on SSD of self-esteem
SSD/MSSD: Squared successive differences/mean squared successive differences, i.e. the mean of the aggregated squared successive differences per participant
WHOQOL-BREF: World Health Organization quality of life questionnaire
